# Modelling typhoid risk in Dhaka Metropolitan Area of Bangladesh: the role of socio-economic and environmental factors

**DOI:** 10.1186/1476-072X-12-13

**Published:** 2013-03-16

**Authors:** Robert J Corner, Ashraf M Dewan, Masahiro Hashizume

**Affiliations:** 1 Department of Spatial Sciences, Curtin University, GPO Box U1987, Perth, Western Australia, 6845, Australia; 2 Department of Geography & Environment, University of Dhaka, Dhaka, 1000, Bangladesh; 3 Institute of Tropical Medicine, Nagasaki University, 1-12-4 Sakamoto, Nagasaki, 852-8523, Japan

## Abstract

**Background:**

Developing countries in South Asia, such as Bangladesh, bear a disproportionate burden of diarrhoeal diseases such as Cholera, Typhoid and Paratyphoid. These seem to be aggravated by a number of social and environmental factors such as lack of access to safe drinking water, overcrowdedness and poor hygiene brought about by poverty. Some socioeconomic data can be obtained from census data whilst others are more difficult to elucidate. This study considers a range of both census data and spatial data from other sources, including remote sensing, as potential predictors of typhoid risk. Typhoid data are aggregated from hospital admission records for the period from 2005 to 2009. The spatial and statistical structures of the data are analysed and Principal Axis Factoring is used to reduce the degree of co-linearity in the data. The resulting factors are combined into a Quality of Life index, which in turn is used in a regression model of typhoid occurrence and risk.

**Results:**

The three Principal Factors used together explain 87% of the variance in the initial candidate predictors, which eminently qualifies them for use as a set of uncorrelated explanatory variables in a linear regression model. Initial regression result using Ordinary Least Squares (OLS) were disappointing, this was explainable by analysis of the spatial autocorrelation inherent in the Principal factors. The use of Geographically Weighted Regression caused a considerable increase in the predictive power of regressions based on these factors. The best prediction, determined by analysis of the Akaike Information Criterion (AIC) was found when the three factors were combined into a quality of life index, using a method previously published by others, and had a coefficient of determination of 73%.

**Conclusions:**

The typhoid occurrence/risk prediction equation was used to develop the first risk map showing areas of Dhaka Metropolitan Area whose inhabitants are at greater or lesser risk of typhoid infection. This, coupled with seasonal information on typhoid incidence also reported in this paper, has the potential to advise public health professionals on developing prevention strategies such as targeted vaccination.

## Introduction

Typhoid fever, an illness caused by a bacterium of the genus *Salmonella*, causes nearly 22 million infections and 200,000 deaths worldwide annually [1]. Salmonella infection in humans can be categorised into two broad types, that caused by low virulence serotypes of *Salmonella enterica* which cause food poisoning, and that caused by the high virulence serotypes *Salmonella enterica typh*i (*S. typhi*), that causes typhoid, and a group of serovars, known as *S Paratyphi* A, B and C, which cause Paratyphoid [2]. Although typhoid infection is infrequent in developed world, it remains a significant threat to the people of developing countries. Regionally, South-central and Southeast Asia has the highest number of cases (>100 per 100,000 people) and fatality rates in the world [3]. A number of cultural, social and environmental factors are associated with the occurrence of typhoid in different endemic settings of which poor quality of life, inadequate provision of safe water and sanitation are found to be the major causes [4-12].

Dhaka, one of the fastest growing megacities in the world, is facing a number of health problems primarily due to rapid population explosion and increased anthropogenic activities. Because of a limited resource-base, it is extremely difficult for local government to ensure adequate public health infrastructure for its ever-increasing population. As a result, water borne diseases have become pervasive in recent times [13]. Diarrhoeal disease, especially cholera and typhoid severely affects the inhabitants of Dhaka [14], particularly those in middle and lower income groups [15]. Due to lack of regular surveillance, an exact estimate of the number of typhoid cases is not possible. However, a few population-based studies have demonstrated that typhoid is a serious public health concern for Dhaka [15-17]. For example, Brook et al. [17] estimated that the overall incidence of typhoid was 3.9 per 1000 persons, disproportionately affecting children [15,16,18]. These studies demonstrate that the perceived burden of typhoid disease could be higher than expected. Contaminated water and food are the common means of transmission [15,16,19] while individual hygiene and poor quality of life are also accountable for typhoid prevalence [18].

The concept of quality of the life (QOL) has recently gained importance for various reasons, including understanding the quality of urban environment [20], as-sessing quality of urban life [21-23], ascertaining people’s satisfaction about their living environment [24,25], evaluating the effectiveness of medical treatments [26] and rehabilitation efforts [27]. QOL is a multidisciplinary construct but is used in the field of public health [28-30] and other areas such as behavioural medicine, political science, psychology, policy making and the planning and management of cities [31,32]. A detailed review of this concept and of its application in different disciplines can be found elsewhere [33,34]. Incorporation of QOL into health research for instance, can provide a number of benefits such as identifying individuals at risk [28] and understanding the constraints of existing health services, thereby allowing improvements in the quality of health services [35]. Historically, micro level data (e.g., household) were used to derive QOL for a given area. At present, macro level studies have become possible because of the capabilities of a spatial information system that allows integration of data from many sources. Using an integrated database together with spatial and statistical techniques, it is now feasible to map the spatial distribution of different aspects of QOL (e.g. environmental, economical, demographic etc.). The outcome from these indicators can subsequently be combined to develop a synthetic QOL [21], urban QOL [22], or environmental quality [20,36]. In addition, neighbourhood quality, a similar type of concept, can also be developed from spatial databases to determine the factors influencing disease incidence [37], and perhaps as an important indicator to identify humans at risk.

Although a generally accepted definition of QOL is not available [33], and it is beyond the scope of this study, a reasonable assumption is that the occurrence of a disease (e.g. typhoid) is the outcome of the quality of socio-environmental factors, the well being or ill being of people and the environment in which they live. Urbanization for instance, is a complex phenomenon and closely linked with the scientific and technological aspects of society, which in turn affects all facets of life and environment [24]. Urban growth, fuelled by population growth and economic development, has two opposing facets. On the one hand, megacities act as engines of economic and social improvement for countries [38], but on the other, improper urbanization directly or indirectly affects the transmission and distribution of disease [39,40]. In addition, rapid urbanization is known to alter the socio-cultural practices of people which in turn have a substantial effect on the prevalence of diseases such as typhoid [2].

As Dhaka is projected to be third largest megacity in the world by 2020 [41], an increase in poverty coupled with an increase in environmental pollution could lead to epidemics of water borne and vector borne diseases in the coming years. For example, unplanned urbanization with little provision of adequate public health infrastructures in Dhaka is already putting hundreds and thousands at risk of gastrointestinal and febrile illness, such as typhoid [14]. Current literature on typhoid infection in Dhaka is based on small populations and conducted in local slums and thus cannot be generalized to the entire metropolitan population [42]. Therefore, updated data are essential to develop effective prevention systems such as vaccination program [43] and to identify members of the population at risk, for public health interventions. Furthermore, a deeper understanding of socio-environmental factors associated with typhoid illness could greatly assist in targeting disease control efforts.

Geographic Information Science (GIS) has become an important tool in understanding the distribution of diseases over space, and such systems have contributed markedly to spatial epidemiological research [44]. In addition, information from earth observing satellites is a powerful data source to complement disease investigation. Many studies have examined vegetation indices, land surface temperature, land use/cover and neighbourhood quality within a GIS to correlate with disease occurrence across the world [37,39,45-49]. Since GIS allows integration of diverse data through geo-coding, causation of disease can be spatially investigated and the output could be used to develop predictive models [44,50]. GIS and spatial statistics have been applied previously to identify typhoid spatial clustering, risk areas and causative factors in the USA and in India [8,51]. These studies demonstrated that spatial techniques are not only powerful for identifying areas and populations at risk but also useful as a guide to health officials for informed decision making.

### Significance

There has been very little work on studying typhoid infection from a spatial standpoint in Bangladesh. This study intends to fulfil the gap by examining the spatial relationships between typhoid and socio-environmental factors derived from satellite remote sensing and census geography in Dhaka Metropolitan Area of Bangladesh. Degrees of health risk will also be estimated by creating a predictable risk model based on the determined factors in spatial analysis.

The techniques used in this study bringing together socio-economic and environmental variables into a Quality of Life Index, capable of application in a wide range of other locations. This study was carried out in the context of an emerging megacity, a class of urban settlement defined by the UN [52] as having more than 10 million inhabitants. Currently, 9.9% of the world urban population lives in 23 megacities which is pro-jected to increase to 37 in 2025 when they are expected to accommodate 13.6% of the world urban population [53]. Further estimates suggest that the number of people living in megacities has increase almost 10 fold in the past 40 years, from 39.5 million in 1970 to 359.4 million in 2011, and could double again by 2025 [53]. The largest increase in urban population is expected to be concentrated in Asia and Africa [54]. These emerging megacities in the developing world share many of the problems that Dhaka faces, and methods developed in this environment will be readily transported.

## Methods

### Study area

**Figure 1:**
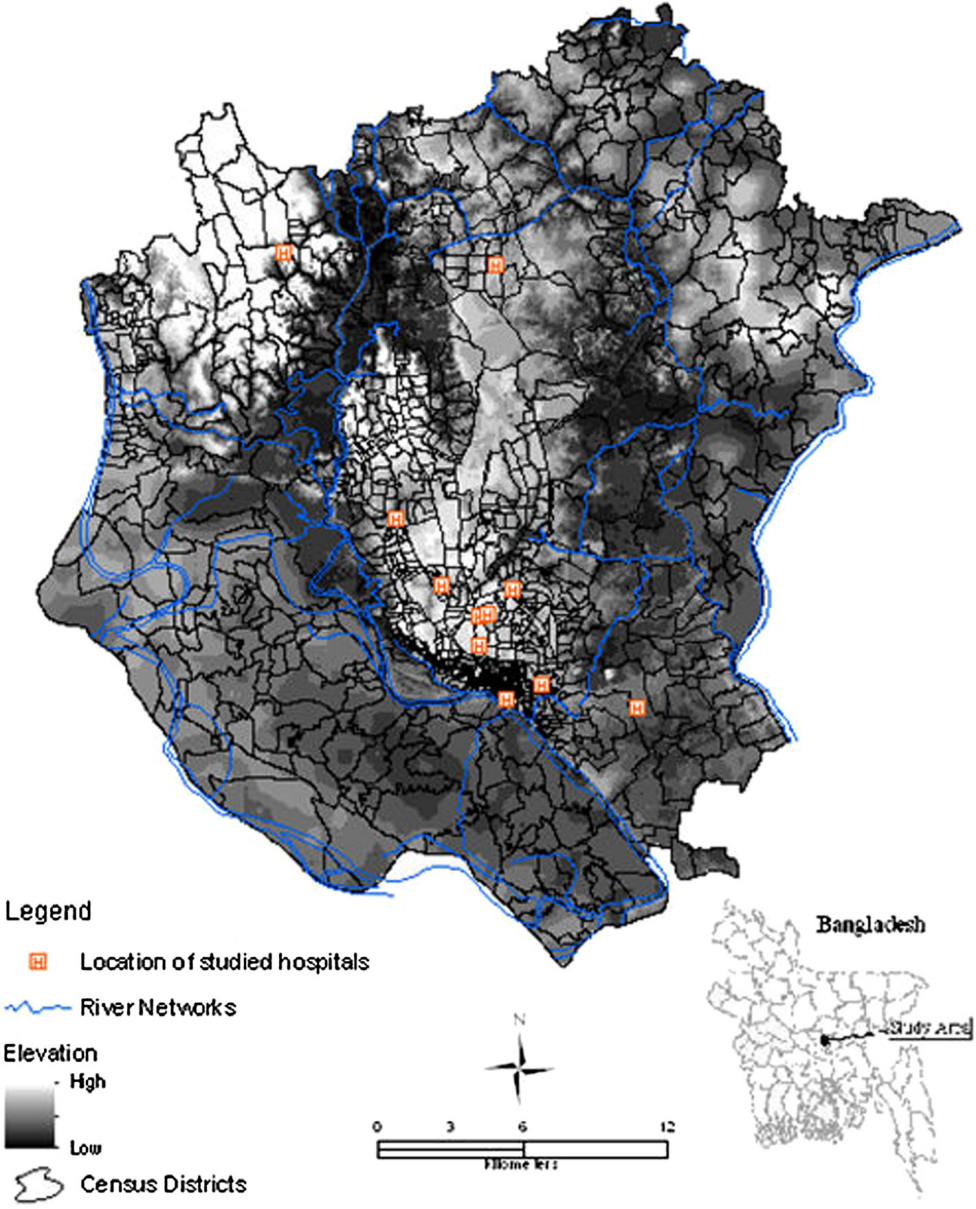
The study area.

The study area was Dhaka Metropolitan Area (hereinafter, DMA) which is in the area of the Dhaka Metropolitan Development Plan (DMDP). The DMA comprises three municipalities, Dhaka City Corporation (DCC), the municipalities of Savar and Tongi, and many unions. DMA is located between 23.61° N and 90.22° E and 23.97°  N and 90.59°  E, and has an area of 878 km^2^ (Figure 1). Based on the 2001 census, the total population of this area was more than 8 million with an average literacy rate of 65% [55]. Topographically, the area is flat with a surface elevation ranging from 1 to 16 meters. The study area is surrounded by five major river systems, namely the Buriganga, Turag, Tongi, Lakhya and the Balu rivers, which flow to the south, west, north, east and northeast, respectively. These rivers are primarily fed by local rainfall but they also receive water from distributaries of the considerably larger Ganges, Brahmaputra and Meghna rivers. DMA has a humid sub-tropical monsoon climate and receives approximately 2000 mm of rainfall annually, more than 80% of which falls during the monsoon, between July and October. Most of the inhabitants in the three municipal areas have access to piped water but outside of these municipalities, drinking water sources may vary (e.g. pond, well, river etc.).

### Typhoid and socioeconomic data

Since no surveillance data on typhoid is available in Dhaka, hospital recorded cases were considered in this study. Multi-year (from 2005 to 2009) typhoid infection data were collected from 11 major health facilities located in the study area (Figure 1). Initially, a standardized form was created to document each case's residence address, demographic and clinical data, date of admission/discharge etc. Using the record room of each hospital, a 30-member data collection team documented the reported cases of typhoid from April to December of 2009. Therefore, this database represents only hospitalized cases and no outpatients were included. All the cases collected refer to diagnosed cases of typhoid at the respective hospital. To avoid data duplication, we first matched data using all the demographic variables and then cross-checked the data against the corresponding date/year in the log books of each hospital. If a case satisfies both of these records, it was then included in the database. We excluded cases residing outside of DMA along with the duplicates (n= 1231). This resulted in a total of 4355 cases pertaining to study area. To minimise error in case mapping, we also cross-referenced each individual case's place of residence with the 2001 census district names by Bangladesh Bureau of Statistics (BBS). When place of residence inconsistencies were found, we used the smallest mapping unit (*mahalla* and *mauza*) since people in the study area are more familiar with local names than administrative units.

The population and socio-economic data were obtained from Bangladesh Bureau of Statistics community series [55] that represents 2001 census information. Since the data was not available digitally, all the variables of interest were first encoded in a spreadsheet and then linked with the appropriate geographic unit by using a series of unique numerical identifiers.

### Ethics statement

All case data collection was carried out with the permission of the Director General of Health, Bangladesh, granted on 10th March 2009. Data collection was carried out in accordance with the standards of the University of Dhaka ethics committee under a permission letter dated 29th March 2009. Data collection took place after this date. Data were anonymised and aggregated at the level of the relevant mapping unit (*mahalla* and *mauza*).

### Geographic and remote sensing data

This study utilises the census tract boundaries of DMA as the mapping units since the use of smaller spatial units has been shown to provide valuable information on the distribution of disease over space [56]. In the absence of up-to-date digital boundary data, we have generated a current census tract boundary shape file using various sources, including the small area atlas from BBS, database from Bangladesh Space Research and Remote Sensing Organization (SPARRSO), the Centre for Environmental and Geographic Information Services (CEGIS) database followed by a number of field visits. Whilst this database was being created, it was found that 25 new census tracts used in the 2001 census, were not identified in the existing spatial data. To identify these, the 1991 census tracts names were first matched with the 2001 census tracts names using the community series of BBS. A hard copy map from BBS, which highlighted the road networks that were used to split the original (1991) census tracts to create new census tracts for 2001 census, was used to digitise the tracts created between decennial censuses. Field visits using a high resolution mobile mapping GPS (Trimble Nomad 800GXE) were used to confirm and correct the road network locations. The final census boundary layer included a total of 1212 polygons of which 441 entities are rural (known as *mauza*/village) and 771 entities were urban (known as *mahalla*/community). Using ArcGIS software (v. 10) [57] we have aggregated all the typhoid cases within each census tract feature. Housing data were obtained from the detailed area plan (DAP) of RAJUK (the capital development authority) and land value data were collected from the respective sub-registry offices.

Apart from the census tract boundaries, the study also utilized a number of remote sensing images to derive spatial information pertinent to the study. A total of ten Landsat-5 Thematic Mapper (TM) scenes, (five adjacent pairs) covering the study area, between 2005 and 2009 were acquired and used. Pre-processing of TM data included georeferencing, mosaicing, subsetting and atmospheric correction [58]. A high spatial resolution GeoEye image from 2010 was also used for various purposes. It was primarily used to develop a slum database and also served to validate land use/cover data. Slum data polygons for the study area were generated through heads-up digitizing supported by field verification in 2010. Initially, 10,159 slum clusters were identified however after field validation, the slum data were consolidated to 9570 clusters distributed across the study area.

The series of Landsat TM data were used to derive land surface temperature (LST), normalized difference vegetation index (NDVI), and a land use/cover map of the study area for the year 2000 was created from a separate image. Only the reflective bands of Landsat TM were used to extract land use/cover of the study area. Using a modified Anderson Level I Scheme [59], land use has been divided into seven categories which are urban, rural settlements, water bodies, wetlands, cultivated land, forest cover and bare land. A hybrid approach (unsupervised-supervised) was used to classify Landsat TM into discrete land use categories [60]. After validation using the high resolution image, the urban category was extracted as a separate dataset. NDVI was derived by using the standard formula (NDVI = (TM3-TM4)/(TM3+TM4)) [61].

LST, a biophysical parameter, for the DMA was derived using the thermal infrared band (TIR) of Landsat TM. Firstly, the digital number (DN) of TIR was converted to spectral radiance [62]. Next, the spectral radiance was converted to blackbody temperature [63]. Using the method suggested by Nichol [64], the temperature data were corrected for surface emissivity. Finally, the images were converted to Celsius units.

Since the remotely sensed, socioeconomic and geographic boundary data had different spatial resolutions and format, they needed to be integrated. Mean NDVI, mean LST, percent urban area, median housing value, housing density and percent slum were calculated using the zonal function of a GIS and aggregated with the census boundary polygons. Total population, per capita land, total literacy rate, percent unemployed, age-specific population, male literacy, female literacy, sources of drinking water and sanitary information were extracted from the census of 2001, and population density was then estimated using the total population and total area for each census district. Due to the skewed distribution of the population density and proportion of slum area datasets, they were log-transformed. Per- capita land and the proportion of each tract occupied by slums were used as surrogates for per capita income since that data was not available in the census. A total of 15 variables related to social, economic, demographic and environmental conditions were defined for analysis as potential predictor variables. Based on the assumption that the typhoid case data were independent between the years and that the geographic variation in the covariates had not changed significantly between years, multiple years of typhoid data were aggregated into one dataset. Table 1 shows the 15 potential predictor variables and the way in which they were derived or computed.

**Table 1:** Demographic, environmental and socioeconomic variables for each census tract

**Variable**	**Derivation**
Total population	From 2001 census records
Population density	Total population / census tract area
Household size (>5)	Number of households in tract with >5 occupants
NVDI	Mean of NDVI from five mosaiced image pairs
Temperature	Mean of LST from five mosaiced image pairs
Percent urban	From Land use/cover classification of 2000 image
Housing density	From RAJUK Detailed Area Plan and tract areas
Per capita land	From 2001 census records and tract area
Total literacy rate	As a percentage from 2001 census records
Percent unemployed	As a percentage from 2001 census records
Percent slum area	From digitised GeoEye image and tract area
Median housing value	Weighted analysis of residential data and census tracts
Households without safe water	As a percentage from 2001 census records
Households that own agricultural land	As a percentage from 2001 census records
Households without sanitation	As a percentage from 2001 census records

### Statistical and geographic analyses

We used geographic information science tools to reveal the spatial pattern of typhoid occurrences in DMA. The number of cases and the population statistics were used to calculate typhoid incidence (expressed as cases per 100,000 persons per year) for each census tract. Temporal patterns of typhoid cases were also investigated and an epidemic curve was prepared based on the annual incidence of typhoid divided by total population for each year multiplied by 100,000 persons, and monthly cases of typhoid infection during the period of 2005–2009. The SQL query tools in ArcGIS were used to determine which census tracts (*mahalla* and *mauza*) had typhoid cases reported in them and to determine the most affected census tracts in each year.

To determine the socio-environmental factors associated with typhoid occurrence in DMA, a range of statistical techniques were employed in this study. First of all, a matrix of pair-wise Pearson's correlation coefficients was computed to determine the interrelationship between the potential predictor variables. Since high correlations existed between the variables, this suggested that regression techniques using all potential variables were not ideal for the development of predictive models using this dataset. To overcome this problem, we employed the Exploratory Factor Analysis tools in the SPSS software suite to reduce data dimensions and redundancy. In this process, all 15 variables are initially considered and the suitability of individual variables and variable combinations to be included in the final set of factors is tested using the Kaiser-Meyer-Olkin (KMO) measure and Bartlett's test of sphericity. The KMO measure of Sampling Adequacy is on a scale of 0–1 and should be greater than 0.50 while the level of statistical significance (*p*-value) for Bartlett's Test of Sphericity should be less than 0.1 [65]. On the basis of these tests, a suite of ten variables was selected to proceed to the next stage of the factor analysis. This next stage uses Principal Axis Factoring to find a set of new axes in rotated multivariate space which are uncorrelated. From these axes a new set of factors are extracted that together explain the majority of the variance of the input datasets. As a general rule only those factors in the rotated multivariate space that have eigenvalues greater than 1 (the variance of individual input variables) should be used. The procedure is somewhat iterative in that at this stage the communality of the input variables needs to be examined. The communality of a variable is the proportion of its variance that is explained by the new factors. Only variables exhibiting communalities >.50 should be included.

In our analysis, the final model using 10 variables resulted in a KMO of 0.785 and a Bartlett’s sphericity significance of 0.000. Table 2 shows the correlation matrix for the 10 variables used. Using the rotated factor loadings, the three principal factors (those whose eigenvalues were greater than 1) were labelled as environmental, economic and crowdedness. A QOL index was then calculated for each census tract using the method of Li and Weng [21], shown in Equation 1, where n is the number of factors used, F_i_ is the factor score for the census tract, and W_i_ is the proportion of variance explained by factor.

(1) $$\mathrm{QOL}=\sum_{1}^{n}F_{i}W_{i}$$

**Table 2:** Correlation matrix between variables

	**TEMP**	**NDVI**	**PURB**	**MHV**	**TLR**	**PCL**	**UNEMP**	**PSLUM**	**PDEN**	**HDEN**
TEMP	1.000									
NDVI	-0.797^**^	1.000								
PURB	0.830^**^	-0.830^*^	1.000							
MHV	-0.536^**^	0.498^**^	0.690^**^	1.000						
TLR	-0.461^**^	0.480^*^	0.618^**^	0.461^**^	1.000					
PCL	0.096^**^	0.047	-0.153^**^	0.307^**^	0.089^**^	1.000				
UNEMP	0.167^**^	-0.123^*^	0.220^**^	-0.358^**^	-0.151^**^	-0.905^**^	1.000			
PSLUM	0.106^**^	-0.050	0.120^**^	-0.038	-0.329^**^	-0.536^**^	-0.532^**^	1.000		
PDEN	0.476^**^	-0.549^*^	0.492^**^	-0.238^**^	-0.284^**^	-0.073^*^	0.121^**^	0.062^*^	1.000	
HDEN	0.562^**^	-0.590^**^	0.561^**^	-0.278^**^	-0.311^**^	-0.076^**^	0.117^**^	-0.049	0.857^**^	1.000

** Statistically significant at 99% confidence level (2-tailed); * statistically significant at 95% confidence level (2-tailed); TEMP: temperature; NDVI: vegetation; PURB: percent urban land; MHV: median house value; TLR: total literacy rate; PCL: per capita land; UNEMP: percent unemployed; PSLUM: percent slum area; PDEN: population density; HDEN: housing density.

The spatial relationships between typhoid and socio-environmental variables in terms of three factors and QOL were then tested separately. As the intention was to develop a spatial predictive risk model of typhoid in DMA, the spatial statistics tools embedded in ArcGIS were used to model the spatial relationships. The Ordinary Least Square (OLS) approach is a global regression model and can be used to determine whether the explanatory variables of interest are free from multicollinearity, coefficients are statistically significant and residuals are not spatially autocorrelated [66,67]. OLS examines variables globally and can be misleading when describing phenomena that vary over space [68]. In contrast, geographically weighted regression (GWR) extends the conventional regression model by incorporating spatial information such as coordinates in the data [69]. It is a measure of local rather than global parameter estimates [70], and effective in determining the underlying local factors for particular spatial patterns. Incorporation of locational information in the GWR model can be expressed as Equation 2 which shows how the OLS model converts to GWR:

(2) $$\begin{array}{c} y=\beta_{0}+\beta_{1}x_{1}+\varepsilon \\ \;\mathrm{becomes}\\ y_{\left(m,n\right)}=\beta_{0\left(m,n\right)}+\beta_{1\left(m,n\right)}x_{1}+\varepsilon_{\left(m,n\right)} \end{array}$$

where, y is the dependent variable, x is the independent variable, β_0_ is the intercept, β_1_ is the regression coefficient, ε is the error term and m, n are the coordinates.

We have used typhoid incidence data as the dependent variable. Three factors extracted from PCA and the synthetic QOL were used as explanatory variables to assess the spatial influences among neighbourhoods [71] using both OLS and GWR models. Since the spatial configuration of features being analysed was non-homogeneous [72], we used an adaptive kernel to solve each regression analysis. In order to understand the model fit and compare the results of the global model with local models [69], the GWR tool was set to determine bandwidth (the number of local observations in each local regression) by minimising the locally corrected Akaike Information Criterion (AICc). Local collinearity, independency and normality of residuals of GWR were further evaluated by inspection of the condition number of the design matrices of the regressions. The largest condition number achieved was 21, smaller than the test value of 30, showed that our model was free from statistical concerns.

Predicted values estimated by GWR model show the spatial distribution of the prevalence of typhoid in DMA. Finally, the population data of each census tract was overlaid with the prevalence map to determine human impact of this prevalence.

## Results

**Figure 2:**
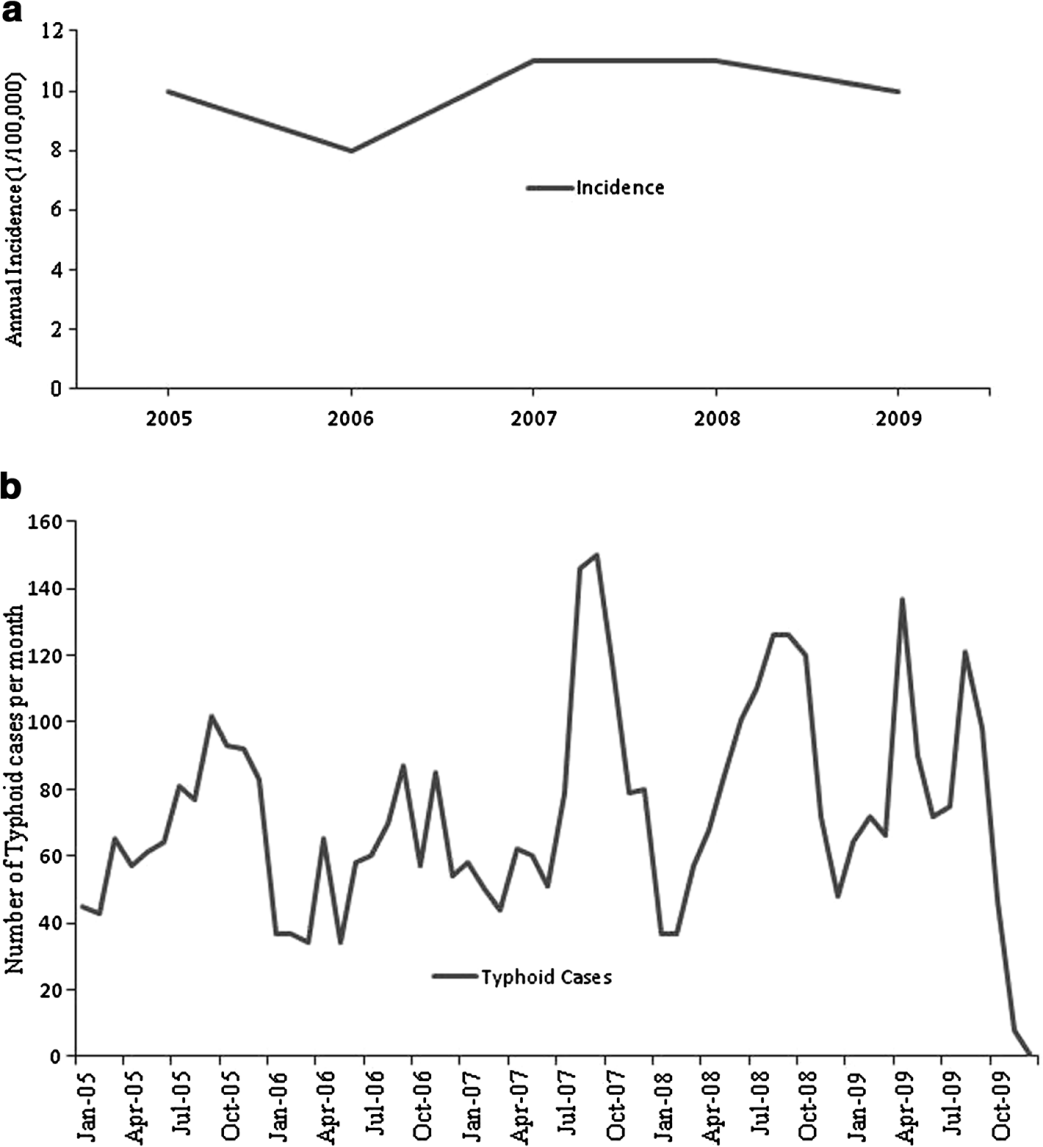
**Temporal distribution of typhoid disease, 2005–2009. a)** Annual incidence rates. **b)** Monthly cases.

**Figure 3:**
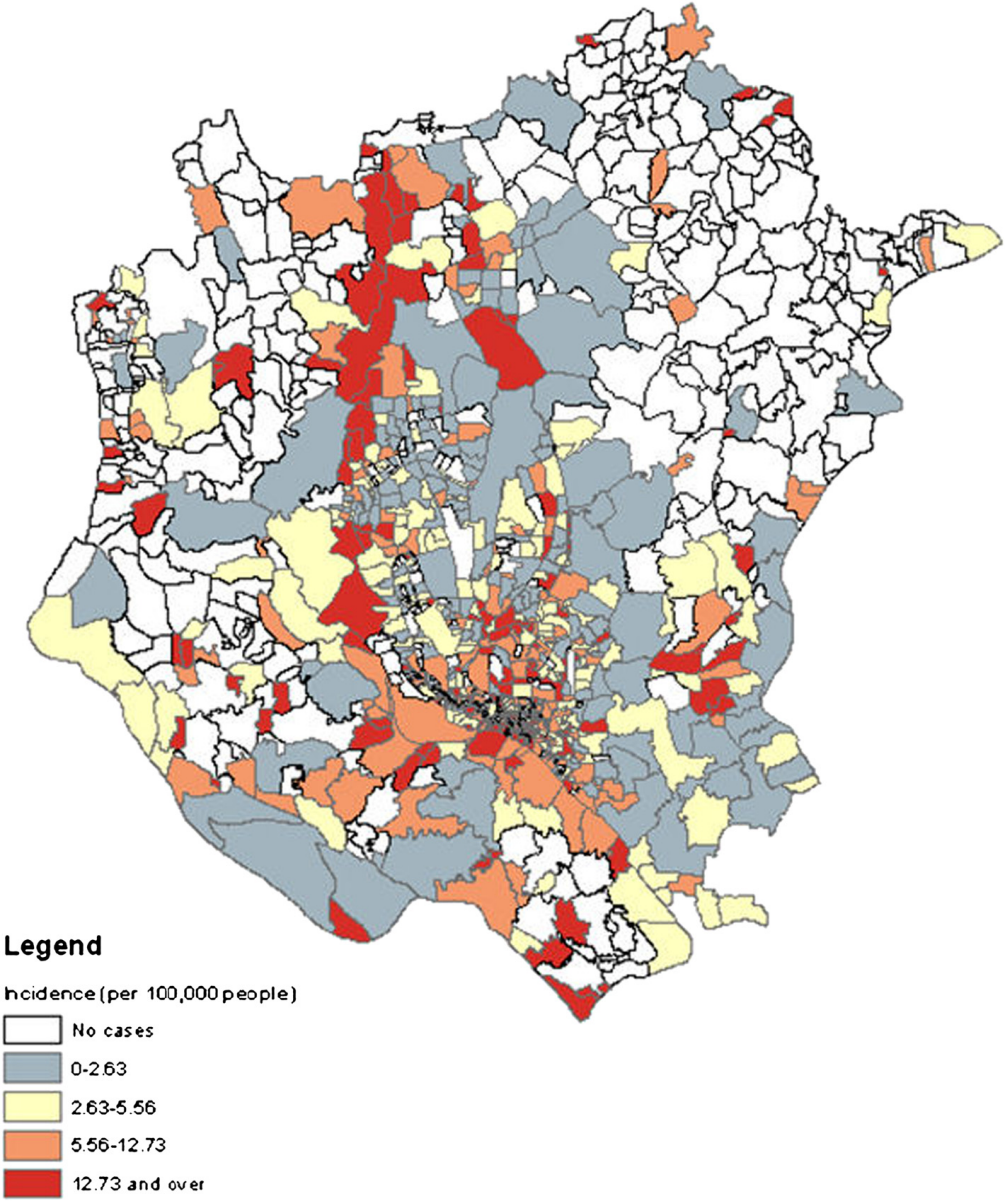
Spatial distributions of typhoid incidence in DMA.

Figure 2 shows the epidemic patterns of typhoid in DMA during the study period, 2005–2009. The annual incidence rate varied from 8 (in 2006) to 11 (in 2007/8) per 100,000 people and the average number of typhoid occurrences in each year was 871. Examination of the monthly distribution of typhoid reveals that the highest cases have fluctuated over the years, July-October being the highest, followed by April-June (Figure 2). Distribution of typhoid cases according to census tract also varied over the years with a maximum in 2008 (Table 3). The highest number of census tracts were infected in the year of 2008 (453) and the maximum number of reported typhoid cases in a neighbourhood was found to be 32 in 2006 (Table 3). Figure 3 shows the spatial pattern of typhoid incidences in DMA. This shows that the spatial distribution of typhoid in the study area is not uniform but on closer inspection it suggests that most of the typhoid cases occurred in the proximity of large water bodies such as rivers and lakes.

**Table 3:** Distribution of typhoid cases by census tract, 2005-2009

**Year**	**Total reported cases**	**Number of census tract infected**	**Highest number of cases in a census tract**
2005	863	410	25
2006	678	358	32
2007	977	408	31
2008	986	453	28
2009	851	410	14
Total	4355	755	130

Analysis of the correlation matrix (Table 2) revealed that the average NDVI in each polygon was negatively correlated with temperature (r = -0.797), with population and housing density (r = -0.549 and -0.590) but positively correlated with percent urban area (r = 0.830) and with economic variables such as median housing value (r = 0.498) and total literacy rate (r = 0.480). Likewise, the percentage of urban area in each tract was positively correlated with housing value (r = 0.690), literacy rate (r = 0.618) and housing density (r = 0.561) but had strong negative correlation with NDVI (r= -0.830), as buildings replace green space. Among the socioeconomic variables, total literacy rate was positively correlated with housing value (r = 0.480) and per capita land (r = 0.307) but negatively correlated with percent slum (r = -0.329), indicating that education attainment is higher in well-off people. On the other hand, population density was significantly correlated with housing density (r = 0.857), implying a degree of overcrowdedness in DMA, which should have substantial impact on the distribution of typhoid. Since these variables depicted high correlation, Principal Axis Factoring was carried out in order to better represent the relationships established among socioeconomic, demographic and environmental factors. Based on the outcome of the rotated factor solutions, Table 4 shows that three factors accounted for 83.24% of the total variance. The first factor explained 46.07%, the second factor 25.55% while the third factor 11.60% of the variance of the input variables. In factor 1, vegetation presents the highest positive loading (loading (L): 0.891) while strong negative loadings on percentage of urban (L: -0.887), temperature (L: -0.782) and population density (L: -0.222), indicate that Factor 1 has clearly characterized positive environmental conditions (factor scores ranged between -2.10 and 3.23). Factor 2 presents strong positive loadings on five socioeconomic variables, including median housing value (L: 0.770), per capita land (L: 0.936), percent of unemployment (L: 0.925), total literacy rate (L: 0.743) and percent of slums (L: 0.753). Hence, Factor 2 can be considered as representing positive welfare or economic condition (scores ranged from -0.80 to 2.22). Factor 3 showed strongest loadings on two variables e.g. population density (L: 0.921) and housing density (L: 0.903) with negative loadings (L:-0.462) for vegetation, since crowded areas are associated with the lowest amounts of green space. Factor 3 scored between -1.37 and 7.89, with higher scores characterizing tracts where very many people live in a small space. As a result, factor 3 was regarded as "crowdedness", a negative factor. Following the method of Li and Weng [21], Equation 1 was rewritten so that the QOL for each tract was derived using Equation 3, below, where F_1_, F_2_ etc. are the factor scores for the individual tracts:

(3) $$\mathrm{QOL}=0.4607{\mathrm{xF}}_{1}+0.2555{\mathrm{xF}}_{2}-0.1160{\mathrm{xF}}_{3}$$

**Table 4:** Factors loading and percentage of variance explained by social and environmental factors

**Components**	**Variance explained**	**Loading**
Factor 1: Environmental	46.07%	
Percent urban		-0.887
Temperature		-0.782
Vegetation		0.891
Factor 2: Economic	25.55%	
Mean housing value		0.770
Total literacy rate		0.743
Per capita land		0.936
Percent unemployed		0.925
Percent slums		0.753
Factor 3: Crowdedness	11.60%	
Population density		0.921
Housing density		0.903
Sum of the variance explained	83.24%	

**Figure 4:**
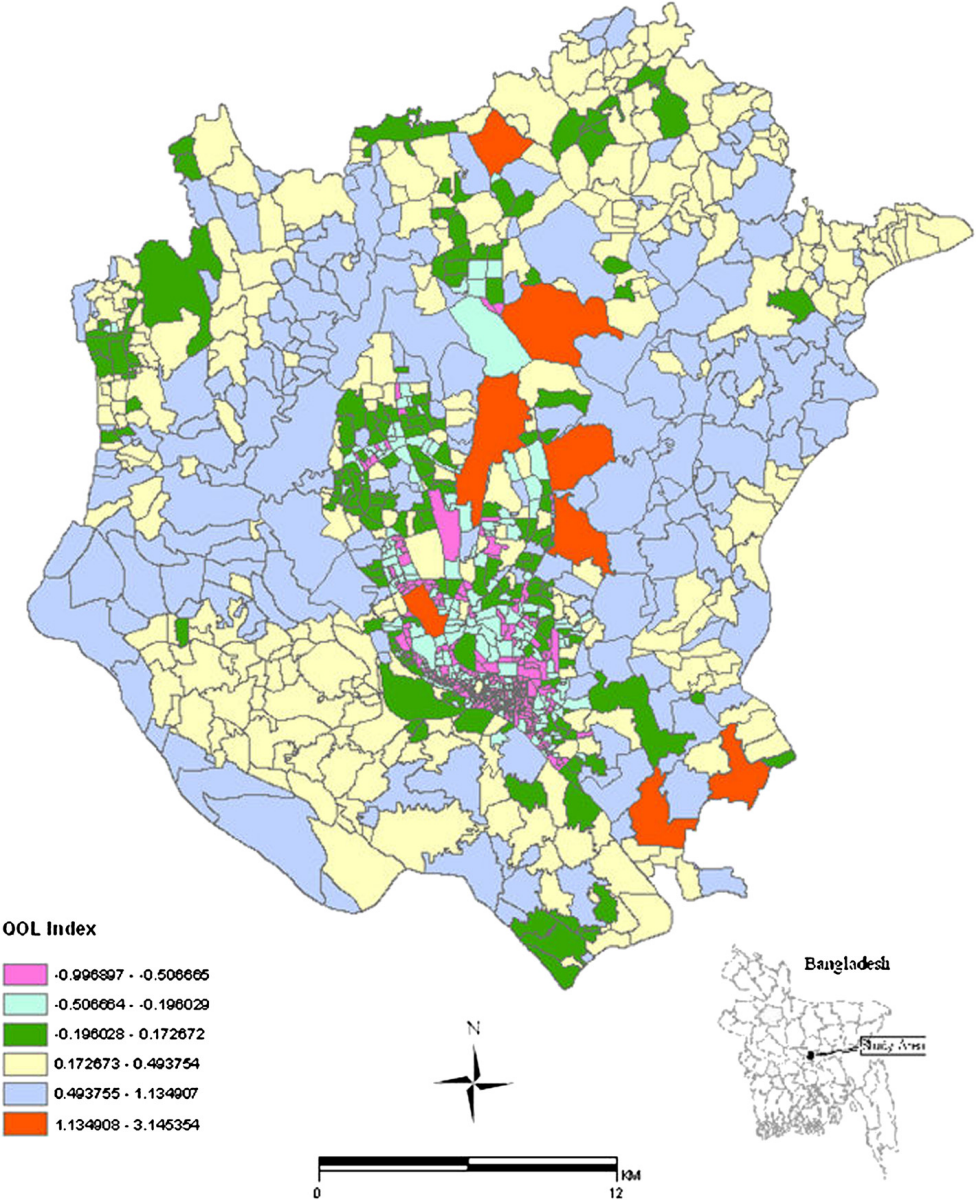
Synthetic quality of life index (QOL) for DMA.

The spatial distribution of QOL scores is presented in Figure 4 and ranges from -0.99 to 3.15. The higher the score the better the quality of life (QOL) is for a particular census tracts. As expected, tract with higher green vegetation and lower population density showed better QOL.

**Figure 5:**
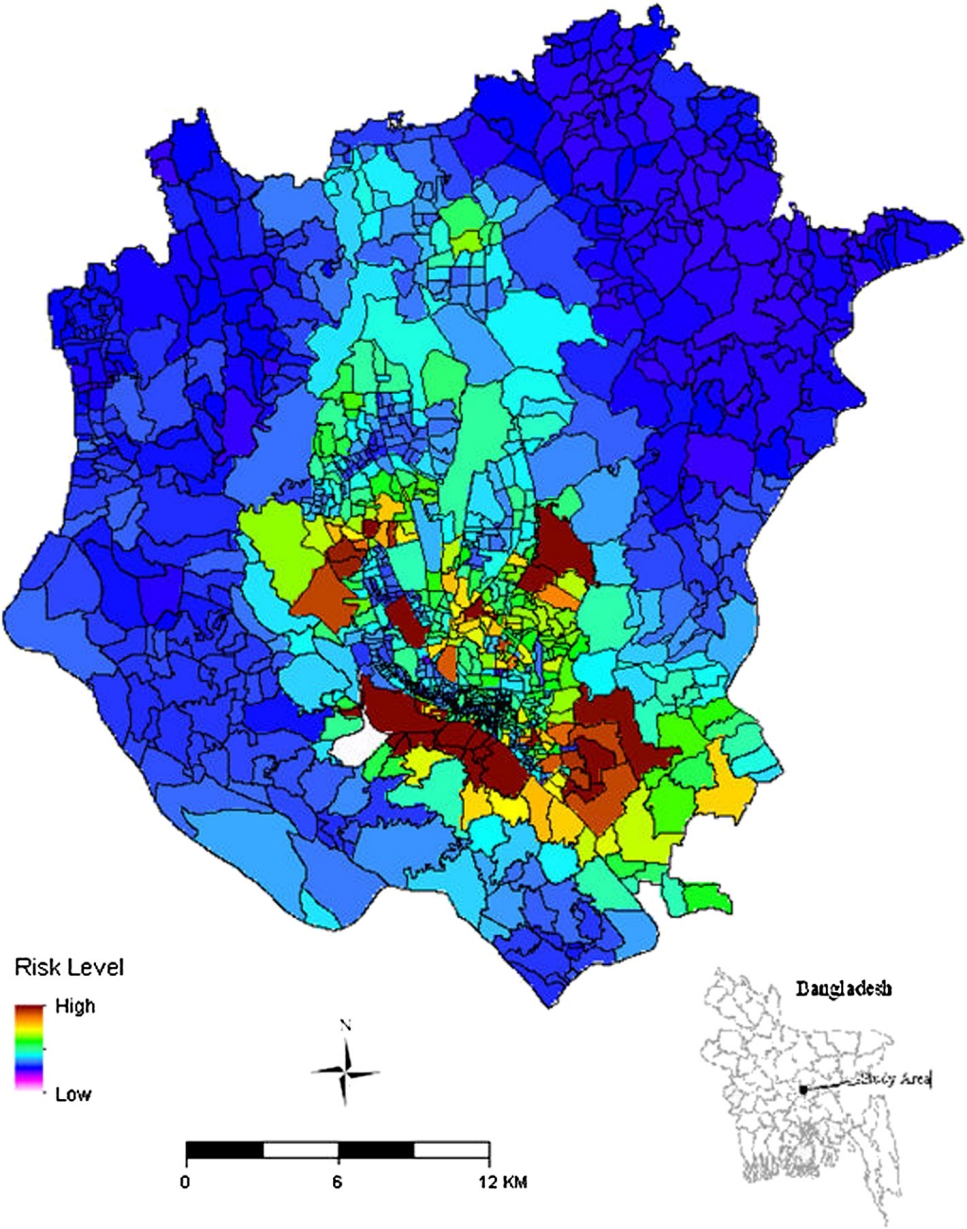
Prediction map of risk of typhoid fever infection based on quality of life index (QOL).

The comparison of the outcomes of global (e.g. OLS) and local (e.g. GWR) models indicated that GWR outperforms the OLS model in terms of AICc and coefficient of determination (r^2^). The relationship between explanatory and dependent variable were tested independently and it was found that QOL alone performed much better than each of the individual factors as an independent variable. AICc values from the OLS model for the independent variables were 8087.46 for factor 1, 7999.20 for factor 2, 8132.79 for factor 3 and 8132.20 for QOL. In contrast, AICc values by GWR were 7590.70, 7597.18, 7671.90 and 7190.24 respectively; showing that for this regression method QOL outperformed the individual factors as a predictor. The coefficient of determination (r^2^), also showed tremendous improvement when GWR was used. For example, the OLS derived r^2^ for factor 1 was 0.037 which increased to 0.633 when using GWR, demonstrating a substantial improvement in the fit of the model to the data. The low r^2^ using OLS was due to the existence of spatial autocorrelation which was understood through the assessment of Moran's *I* statistics. For instance, Moran's *I* of standard residuals of the OLS results for factor 1, 2, 3 and QOL were 0.341, 0.351, 0.388 and 0.382 respectively, indicating that a local model was needed to solve the regression equation. Full details of the relative regression quality measures are shown in Table 5. Since QOL showed the highest correlation with the incidence of typhoid (r^2^ =0.73), predicted values from a GWR model using QOL as the independent variable were used to develop a typhoid prevalence map (Figure 5). We have expressed this as risk although the quantitative units are arbitrary and negative risk does not imply protection. Risk was categorised for further analysis into High, Moderate and Low risk (Table 6). Overlaying the predictive model with population data demonstrated that 9.16% population of DMA are at high risk, 44.01% people are at moderate risk and 46.83% are at low risk of typhoid.

**Table 5:** Comparison of OLS and GWR results

**Explanatory variable**	**OLS**		**GWR**	
	**r**^**2**^	**AICc**	**r**^**2**^	**AICc**
Factor 1	0.037	8087.46	0.606	7590.70
Factor 2	0.105	7999.20	0.532	7597.18
Factor 3	0.001	8132.79	0.633	7671.90
QOL	0.001	8132.20	0.731	7190.24

**Table 6:** Cut off values for risk categories

**Risk score**	**Risk category**
<4.62	Low risk
4.62 to 16.81	Moderate risk
16.81>	High

## Discussion

Using five years of reported typhoid data with spatial analytical techniques, this study is the first to explore the relationships between socio-environmental variables and typhoid occurrences in DMA. In the absence of regular surveillance, findings from this study in DMA not only provide insight about spatial-temporal patterns of typhoid but also suggested the socio-environmental factors associated with the disease.

Typhoid disease is very common in South Asia owing to the fact that this is one of the most impoverished regions of the world where poverty is consistently rising and a larger portion of population is lacking potable water and safe sanitation. A temporal epidemic curve reveals that yearly typhoid incidence rate was 8–11 persons per 100,000 people with the peak incidence rate in the period under consideration occurring in 2007/8. Monthly records demonstrated that almost half of the reported cases had occurred during the monsoon (July-October), indicating a distinct seasonal pattern. This finding supports an earlier clinical-based study conducted in the same area [15]. Environmental factors are known to have impact on the distribution and transmission of typhoid in other endemic settings. Rainfall for instance, substantially affected the occurrence of typhoid by increasing the faecal contamination in the water supply in Pakistan [73], and the transmission of typhoid bacterium is to some extent influenced by rainfall, particularly in low lying areas where people rely on surface water for their daily needs, including drinking and domestic purposes [9]. When natural runoff drains and transports rubbish, including human wastes to the surrounding water bodies during the monsoon, surface water becomes heavily contaminated, resulting in a higher number of cases of typhoid [8]. Since water logging and flooding become pervasive during the monsoon in DMA, contamination of surface water [74] and tube wells [75] by flooding are likely to result in a peak incidence at that time. Furthermore, flooding, either natural or caused by human modification of the land surface could lead to the occurrence of typhoid [11], particularly in many wet locations like DMA. Not all the census tracts in the study area are equally susceptible to typhoid infection; generally areas with higher population density and inadequate provision of health infrastructure suffer from higher cases of typhoid infection, corroborating the results of an earlier study by Naheed et al. [16].

The spatial pattern of typhoid incidence indicated significant variation of the disease distribution in DMA (Figure 3). A close visual inspection of the incidence map suggested that census districts closer to large water bodies (e.g. river networks and lakes) are highly vulnerable to elevated incidence rate. This finding can be explained by the fact that both surface and groundwater water quality get severely degraded due to increased anthropogenic activities in DMA, which may have significant impact on the transmission and distribution of typhoid. In addition, low income people in the study area use surface water for cooking, bathing and other purposes. Consequently, a reasonable assumption is that contamination of these water bodies could directly influence the disease dynamics in the communities which is in agreement with a study conducted in Indonesia [12]. As *Salmonella* bacteria can survive in water for days [76], contaminated surface water such as sewage, freshwater and groundwater could act as etiological agents of typhoid [77]. It was generally observed that communities living in the proximity of the rivers Buriganga, Turag, and Balu had an elevated risk of typhoid compared with communities in other locations. These three rivers have found to have extreme pollution loads throughout the year in terms of coliform counts and other physio-chemical parameters [78-80], hence the probability of increasing of the disease burden is warranted. Also, risk factors investigations for typhoid have substantiated that all sources of drinking water, including piped water is highly contaminated in Dhaka [15,19]. This accords with a study in Tajikistan [81] where contamination of piped water was found to have significant association with the occurrence of typhoid. These studies indicated that contaminated surface and piped water in DMA could amplify the likelihood of water borne infection among people living in that area. The transmission dynamics of typhoid in relation to water quality therefore remains a very promising area to explore.

A number of environmental, socioeconomic and demographic variables were combined through Principal Axis Factoring to classify each census tract according to three principal factors (e.g. environmental, economic and crowdedness), and to use the resulting score for risk area identification. The results demonstrated that QOL could serve as an important indicator as it was able to explain 73% variance in the model as an independent factor. This finding is in agreement with Khormi and Kumar [37] who found that neighbourhood quality provided the highest coefficient of determination in explaining the incidence of dengue disease in Saudi Arabia. Out of three factors extracted, factor 3 (e.g., so-called crowdedness index) had the highest coefficient of determination (r^2^=.63) followed by factor 2 (r^2^=.53) and factor 1 (r^2^=.60) based on individual GWR analyses, implying that population density, large households size and housing density have substantial impact on typhoid incidence. The study statistically substantiates the concept that areas with low risk of typhoid have a low mean population density (49069/km^2^), those with medium risk had a medium mean population density (633387/km^2^) while high risk areas had the highest mean population density (67464/km^2^). Similarly, literacy rate, water sources, unemployed population, percentage of slum area, sanitary facilities were higher in low risk areas than that of medium and high risk areas, illustrating the effect that socioeconomic status, water sources and sanitary facilities have on typhoid distribution in DMA [16]. Crowdedness is regarded a sign of depressed socioeconomic conditions that facilitate person to person transmission [8] by sharing the same plate for food [11], cups and mugs for drinking, by being in contact with the infected person [82] or by residing in the same place [10]. In addition, lack of education could put individuals at high risk as it is often related to poverty, poor housing condition, inadequate provision of safe sanitation and unemployment [8,12,18,83]. We have also found that of the areas at high risk areas, 72.73% had low QOL, 18.19% medium QOL and 9.08% presented high QOL. Thus, it may be assumed that unplanned urbanization, higher population density, lack of critical urban infrastructures, particularly in DMA, have a considerable impact on the transmission and distribution of typhoid fever. While an advantage of the Principal Axis Factoring is that it reduces the complexity of correlated data and allows combining diverse data into fewer factors, a potential problem however is that it could lead to the loss of information through generalization [84] and a loss of direct causal relationships to raw predictor variables.

Spatial relationships were determined through global and local models, and the study recognized the efficacy of the GWR model to provide useful information about geographical heterogeneity. The GWR performed much better because the global model assumes the relationship between explanatory and dependent variables are consistent, and provides an average state of the phenomena being studied. The local model on the other hand, assumes the relationships are non-stationary. Since AICc is an effective way of comparing two models [85], the considerable difference in that measure implied an important improvement in the model fit [66]. The results of r^2^ and AICc indicated GWR was a better model to predict typhoid risk in DMA.

Spatial statistics is gaining renewed interest as a means to attribute disease association and risk. Even though GWR has long been used in various studies including public health, crime and demography [86-89], there are some limitations of the model. One of such problems is the choice of appropriate kernel type and bandwidth to which the model is sensitive [90]. Another notable problem is that the non-linear terms cannot be added to GWR models [69].

This study has a few limitations. First of all, the disease data that were acquired from hospitals may have underestimated or overestimated typhoid records. Because the data were historical records and documented from the record room of each hospital, we had no valid method to ascertain repeated hospitalizations of an individual patient. In addition, hospital-based surveillance may underestimate actual population at risk because only severely sick people tend to get admitted for treatment. Secondly, we only consider 11 major health service providers, the majority of which were public hospitals. The study could be improved by including data from private clinics where most of the affluent people seek health services. On balance, we believe that we have an underestimate of the occurrence. We do not believe that this affects the validity of our results since we have been able to develop a predictive model using what is effectively a sample of unknown size drawn from the true population of occurrences. Thirdly, we also could not separate cases into typhoid and paratyphoid groups. Isolation of these two types would allow us to estimate the disease dynamics and identify the most prevalent typhoid types in DMA. The etiology of the two diseases is similar but the morbidity rates are not. Again we believe this does not affect the validity of our results since we are dealing with disease occurrence, not disease outcome. Fourthly, a new method is needed to overcome the problems associated with GWR such as mixed geographically weighted regression proposed by Mei et al. [91]. Finally, water source and sanitation data of each census tract could greatly improve future study since these variables are known to have considerable impact on the occurrence of typhoid.

Despite the limitations listed above, the major strength of this study is the derivation of the first regional risk map of typhoid infection which rigorously investigated a fine-scale spatial distribution of typhoid and its socio-environmental determinants. Moreover, the study determined that QOL could be an important indicator in identifying populations at risk of typhoid in a rapidly urbanizing megacity where high quality data is lacking. Although vaccination is available to prevent typhoid infection, it cannot be an alternative to sound environmental health infrastructures [92]. Furthermore, DMA is likely to encounter rapid urban growth and more intense rainfall, driven by climatic change, in the coming years. These changes may put more people at risk of typhoid. Therefore, this study underscores the necessity of appropriate policies as well as critical public health infrastructures to curb the future spread of water borne diseases.

## Conclusions

Spatial methods were utilized to explore the spatio-temporal distribution of typhoid and associated socio-environmental factors obtained from diverse sources. Using census tracts as the spatial unit, the study examined various socioeconomic, demographic and environmental parameters to develop a quality of life index (QOL). Derived indices were analysed through ordinary least square (OLS) and geographically weighted regression (GWR) techniques, to account for local variations of the predictors. It was found that QOL served better to complement the understanding of phenomenon that had important spatially varying relationships. The typhoid risk map developed in this study can guide public health officials to develop an early warning system for the prevention and control of water borne disease in DMA or elsewhere.

## Competing interests

The authors declare that there are no competing interests.

## Authors’ contributions

RJC and AMD designed the study and acquired the Remotely Sensed (RS) data. AMD oversaw the acquisition of the typhoid data and collected and tabulated the socioeconomic data. RJC and AMD processed the RS and disease and socioeconomic data, derived and extracted the environmental variables, carried out the statistical analysis and drafted the manuscript. MH assisted in the construction of the statistical analysis framework as well as in drafting the manuscript. All authors read and approved the final manuscript.
